# Curcumin-induced HDAC inhibition and attenuation of medulloblastoma growth *in vitro *and *in vivo*

**DOI:** 10.1186/1471-2407-11-144

**Published:** 2011-04-18

**Authors:** Seung Joon Lee, Candice Krauthauser, Victoria Maduskuie, Paul T Fawcett, James M Olson, Sigrid A Rajasekaran

**Affiliations:** 1 Nemours Center for Childhood Cancer Research, Alfred I. duPont Hospital for Children, 1701 Rockland Road, Wilmington, DE 19803, USA; 2 Biomedical Research, Alfred I. duPont Hospital for Children, Wilmington, DE 19803, USA; 3 Fred Hutchinson Cancer Research Center, 1100 Fairview Avenue North, Seattle, WA 98109, USA

## Abstract

**Background:**

Medulloblastoma is the most common brain tumor in children, and its prognosis is worse than for many other common pediatric cancers. Survivors undergoing treatment suffer from serious therapy-related side effects. Thus, it is imperative to identify safer, effective treatments for medulloblastoma. In this study we evaluated the anti-cancer potential of curcumin in medulloblastoma by testing its ability to induce apoptosis and inhibit tumor growth *in vitro *and *in vivo *using established medulloblastoma models.

**Methods:**

Using cultured medulloblastoma cells, tumor xenografts, and the Smo/Smo transgenic medulloblastoma mouse model, the antitumor effects of curcumin were tested *in vitro *and *in vivo*.

**Results:**

Curcumin induced apoptosis and cell cycle arrest at the G2/M phase in medulloblastoma cells. These effects were accompanied by reduced histone deacetylase (HDAC) 4 expression and activity and increased tubulin acetylation, ultimately leading to mitotic catastrophe. In *in vivo *medulloblastoma xenografts, curcumin reduced tumor growth and significantly increased survival in the Smo/Smo transgenic medulloblastoma mouse model.

**Conclusions:**

The *in vitro *and *in vivo *data suggest that curcumin has the potential to be developed as a therapeutic agent for medulloblastoma.

## Background

Brain tumors are the second most frequent malignant tumors in children and are generally associated with a worse prognosis when compared with other common pediatric cancers [1]. Among pediatric brain tumors, medulloblastoma is the most common malignant form [2]. Despite recent improvements in survival rates, medulloblastoma is incurable in about a third of patients, and survivors undergoing current treatment suffer from serious therapy-related side-effects [3]. Most medulloblastomas are thought to originate from cerebellar granule neuron precursors (CGNPs) [4], and several signaling pathways have been implicated in medulloblastoma formation including aberrant activation of WNT, sonic hedgehog (Shh), and epidermal growth factor receptor (EGFR) signaling cascades. Consequently, several therapeutic strategies, such as monoclonal antibodies and small molecule inhibitors, have been employed to target these pathways and succeeded in eradicating spontaneous medulloblastoma in transgenic and transplantation mouse models [5]. However, while these agents might have limited to no side effects in adults, in juvenile mice, even transient exposures to a Shh pathway inhibitor resulted in permanent defects in bone development [6], impeding the therapeutic potential against pediatric cancers. Thus, it remains a challenge to identify safe and effective treatment options for pediatric brain tumors, such as medulloblastoma.

Curcumin, also known as diferuloylmethane, is a major component of the spice turmeric derived from the plant *Curcuma longa*. It has been used widely in India and other parts of Southeast Asia as a spice and a medicine with anti-inflammatory and anti-oxidant properties. Recently, curcumin has been highlighted as a potent anti-cancer agent, with chemopreventive and chemotherapeutic potential with no discernible side effects. Curcumin inhibits the proliferation of diverse tumor cells in culture, prevents carcinogen-induced cancers in mouse models, and impedes the tumor growth in various xenotransplant and orthotransplant mouse models [7,8]. Therapeutic efficacy of curcumin by itself or in combination with other drugs is in phase I/II clinical trials against several adulthood tumors such as colorectal, liver, pancreatic, and prostate cancer and against multiple myeloma [7,8]. The possible chemotherapeutic effects of curcumin are now being well-accepted in adulthood cancers. Curcumin has been used safely as a dietary component for centuries and, thus, may prove to be a potentially safer drug alternative in pediatric cancers.

Most importantly, curcumin has the ability to cross the blood-brain barrier (BBB) [7,9-11]. BBB is a specialized system of brain microvascular endothelial cells that separates the central nervous system from the peripheral blood and serves to supply brain tissue with nutrients, to protect the neuroparenchymal microenvironment, and to shield the brain from potentially toxic substances in the blood, including therapeutic drugs. Consequently, the failure of treatment in many instances is not due to an intrinsic lack of potency of the drugs, but instead due to the BBB, which impedes efficient drug delivery [12,13]. Since curcumin can cross the BBB [7,9-11], it may thus prove effective for chemotherapy for pediatric brain tumors.

Epigenetic modifications including acetylation of histones and non-histone proteins play a central role in the development of human cancers [14,15]. The acetylation status of proteins is determined by histone deacetylases (HDACs) and histone acetyltransferases (HATs) that remove and add acetyl groups to lysine residues, respectively. By removing acetyl groups from histones, leading to chromatin condensation, HDACs can act as transcription repressors that selectively alter gene transcription. In addition, HDACs have many non-histone protein substrates such as transcription factors, hormone receptors, signaling mediators, chaperones, and cytoskeletal proteins, which regulate cell proliferation and cell death [16,17]. At present, 18 HDAC isoforms are known and classified into four groups based on their structural homology: the classical Zn^2+^-dependent class I, class IIa, class IIb HDACs and the NAD^+ ^dependent sirtuins (class III), and HDAC11 (class IV) [18]. The ubiquitously expressed class I HDACs are the best-characterized of these proteins. With their primarily nuclear localization, they are crucial for transcriptional repression and epigenetic landscaping. Class II HDAC family members have a more tissue-specific expression pattern, and class IIa members are primarily expressed in heart, smooth muscle, and brain. HDACs are considered promising targets in drug development for cancer therapy [19-21]. HDAC inhibitors can cause cell cycle arrest and induce growth arrest, differentiation, or apoptosis *in vitro *and *in vivo*. The first clinical trials have shown their potential as therapeutics for hematological and solid epithelial tumors in adult patients [22]. In neuronal cells, HDAC inhibitors have yielded conflicting results. For example, HDAC inhibition blocks neuronal loss in a mouse model of Huntington's disease and in Drosophila, suggesting that HDAC inhibitors are neuro-protective [23-25]. In cerebellar granule neurons, pharmacological inhibition of HDACs induced apoptosis [26-29], suggesting that individual HDAC members may have distinct and sometimes opposing roles, given the cellular context [30].

Curcumin interacts with a wide variety of proteins to modify their expression and activity, ultimately inhibiting cell proliferation, invasion, angiogenesis, and metastasis of different types of cancers. While the primary molecular targets and mechanisms of curcumin action remain to be determined, curcumin has been shown to induce apoptosis in a wide variety of cell lines and inhibits tumor growth in *in vivo *models of various cancers [7,8]. We found that curcumin induces cell cycle arrest and elicits apoptosis in medulloblastoma cells. Inhibition of cell cycle progression by curcumin was accompanied by altered organization of mitotic spindle microtubules, probably due to increased tubulin acetylation. Consistent with increased tubulin acetylation, curcumin inhibited HDAC activity and repressed HDAC4 expression in medulloblastoma cells. Although curcumin-induced cell death in medulloblastoma cells has been reported in earlier studies [31,32], we show for the first time that curcumin reduces tumor growth in medulloblastoma xenografts and increases survival in the Smo/Smo transgenic mouse model of medulloblastoma. Thus, curcumin may be a useful for children with medulloblastoma.

## Methods

### Cell lines and reagents

The human medulloblastoma cell lines DAOY, D283 Med, and D341 Med were obtained from the American Type Culture Collection (ATCC, Manassas, VA) and cultured in MEM supplemented with 10% (DAOY, D283 Med) or 20% (D341 Med) fetal bovine serum, glutamine and penicillin/streptomycin in a humidified, 5% CO_2 _atmosphere at 37°C. The DAOY cell line stably expressing tdTomato was generated by transfecting ptdTomato-N1 (Clontech, CA) into DAOY cells followed by selection with 500 μg/ml of G418 for 2 weeks. Cells were then diluted serially for clonal isolation and ptdTomato positive clones were used for xenograft studies.

Curcumin and antibodies against actin and β-tubulin were purchased from Sigma-Aldrich (St. Loius, MO). Antibodies against acetylated tubulin, cleaved Caspase3, cleaved PARP, GAPDH, NFκB, HDAC2, HDAC4, HDAC5, HDAC7, phospho-HDAC4 (S246)/HDAC5 (S259)/HDAC7 (S155), and horseradish peroxidase (HRP)-conjugated secondary antibodies were obtained from Cell Signaling Technology (Danvers, MA). Antibodies recognizing acetyl histone was purchased from Millipore (Billerica, MA) and HDAC6 antibody from Abcam (Cambridge, MA). Antibody against cyclin B1 was obtained from Santa Cruz Biotechnology (Santa Cruz, CA). Alexa 488™-conjugated secondary antibody and phalloidin-Alexa 546™ were obtained from Molecular Probes/Invitrogen (Carlsbad, CA). Pan caspase inhibitor z-VAD-FMK was purchased from Promega (Madison, WI).

### Cytotoxicity assay

LDH levels were determined using the Non-radioactive Cytotoxicity Kit (Promega, Madison, WI) according to manufacturer's instructions. Cells plated in a 24-well plate were incubated with different concentrations of curcumin for various lengths of time as indicated. To obtain the released LDH, media were collected and cell debris was removed via brief centrifugation. Viable cell LDH was collected after re-adding 1ml of fresh serum-free medium. Cells were lysed by freezing for 15 minutes at -70°C followed by thawing at 37°C. The medium was collected and cleared from cell debris using centrifugation. The relative release of LDH was determined as the ratio of released LDH versus total LDH from viable cells. Assays were performed twice in triplicate.

### Immunoblotting

Cell lysates were prepared in a buffer containing 20 mM Tris (pH 7.5), 150 mM NaCl, 1 mM EDTA, 1 mM EGTA, 0.1% Triton X-100, 2.5 mM sodium pyrophosphate, 1 mM b-glycerolphosphate, 1 mM sodium vanadate, 1 mM phenylmethylsulfonyl fluoride and 5 mg/ml of antipapain, leupeptin and pepstatin, sonicated and briefly centrifuged. Protein concentrations of the supernatants were determined by the DC protein assay (Bio-Rad, Hercules, CA). Equal amounts of protein were resolved by SDS-PAGE and transferred to nitrocellulose. The membranes were blocked in 5% non-fat milk in tris-buffered saline with 0.1% Tween 20 (TBST) and then incubated overnight at 4°C with primary antibodies diluted in 5% bovine serum albumin/TBST. After incubation with HRP-conjugated secondary antibodies in 5% non-fat milk/TBST, the protein bands were visualized by Enhanced Chemiluminescence Plus (GE Healthcare, Piscataway, NJ).

### Immunofluorescence

Cells grown on glass coverslips were incubated with curcumin as indicated and fixed with either ice-cold methanol (β-tubulin, acetylated tubulin) or 4% paraformaldehyde (cleaved caspase-3, HDAC4) with subsequent permeabilization with saponin (Sigma-Aldrich). For analysis of mitotic cells, DAOY cells were synchronized by incubation with 2 mM thymidine for 18 hours. Subsequently, after the block was released for 3 hours, cells were arrested in prometaphase with 100 nM nocodazole for 8 hours. The block was then released in the presence of DMSO or curcumin as indicated, and the cells were fixed as described above. Primary antibodies were diluted in PBS with 1% bovine serum albumin (PBS-BSA) and incubated overnight at 4°C. Samples were then incubated with Alexa 488™- or Alexa 546™-conjugated secondary antibodies and mounted in Prolong Gold (Invitrogen). DNA was visualized with TO-PRO3 (Invitrogen) after incubation with RNase A. Images were acquired with a Leica TCS SP5 laser-scanning confocal microscope and LSM software (Leica Microsystems, Mannheim, Germany).

### Cell cycle analysis

DAOY cells were treated with curcumin for indicated times, harvested, fixed in cold 70% ethanol, and stored overnight at -20°C. DNA was stained with 100 mg/ml propidium iodide (PI) and 20 mg/ml ribonuclease A in hypotonic citrate buffer. Samples were analyzed on an Accuri C6 flow cytometer system (Accuri Cytometers, Ann Arbor, MI) as described [33]. Interference from curcumin auto-fluoresence was not observed with the parameters used to acquire the profiles.

### HDAC activity assay

HDAC activity was measured with the fluorometric HDAC Activity Assay Kit (Abcam, Cambridge, MA) according to manufacturer's protocols. Briefly, cells were incubated with increasing concentrations of curcumin for 3 hours and then lysed with a buffer containing 50 mM HEPES, 150 mM NaCl, and 0.1% Triton X-100 supplemented with protease inhibitors. The cell lysates were sonicated, cleared, and incubated with assay buffer containing the HDAC substrate [Boc-Lys(Ac)-AMC] for 30 min at 37°C. The reaction was terminated, and the fluorescence intensity was measured in a fluorescence plate reader with Ex. = 350-380 nm and Em. = 440-460 nm.

### In vivo studies

Female hairless SCID mice (Charles River Laboratories, Wilmington, MA), 5-6 weeks old, were injected subcutaneously with 7 × 10^6 ^viable DAOY cells or tdTomato-expressing DAOY cells with matrigel. Tumors were allowed to grow for 30 days before oral administration was begun. Corn oil or curcumin dissolved in corn oil (1 mg/kg body weight) was delivered daily by oral gavage to each group (8-12 mice per group). The tumor size was measured twice a week with a caliper, and tumor volumes were calculated according to the formula length × width × depth × 0.5. Mice with weight loss of ≥15% of the initial weight or a tumor volume ≥2,000 mm^3 ^were euthanized. Tumors were harvested, and tumor lysates were prepared in buffer containing 10 mM Tris-HCl, 150 mM NaCl, 1 mM EDTA, 0.1% Triton X-100 supplemented with phosphatase and protease inhibitors.

Fluorescence signals from tumor xenografts of tdTomato-DAOY cells were acquired once a week with a Kodak *In Vivo *Multispectral FX PRO imaging system (Carestream, Woodbridge, CT) using the following settings: Ex. 550 nm, Em. 600 nm, no binning, f/stop 2.8, focal plane 13.1 mm, field-of-view 119.1 mm.

Smo/Smo transgenic mice [34] were treated with curcumin or corn oil daily by oral gavage from the point of weaning (9 mice per group). Treatment was continued until clinical manifestation of the disease, when animals were euthanized and tumor tissues were collected for analysis. Animal experiments were performed according to the NIH Guide for the Care and Use of Experimental Animals and approved by our Institutional Animal Care and Use Committee. All animals were given free access to water and feed.

### Statistical analysis

Data are presented as mean ± SD unless otherwise indicated. Differences between means of the two groups were analyzed with the use of a two-tailed unpaired Student's t-test or two-way ANOVA test. Survival curves for Smo/Smo transgenic mice were analyzed using the non-parametric Kaplan-Meier method. When required, P values are stated in the figure legends.

## Results

### Curcumin induces apoptosis in medulloblastoma cells

**Figure 1:**
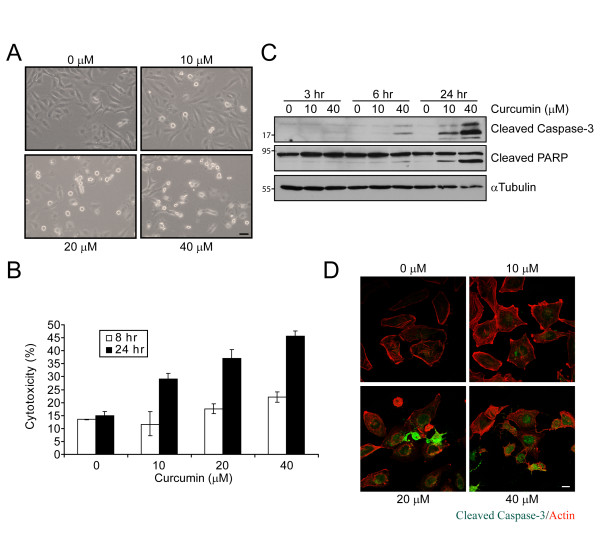
**Curcumin induces apoptosis in medulloblastoma cells**. A. Phase contrast images of DAOY cells incubated with indicated concentrations of curcumin for 16 hours. Bar, 100 μm. B. LDH release as measure of the cytotoxic effect of curcumin. C. Immunoblot of cleaved caspase-3 and PARP in curcumin-treated DAOY cells. α-tubulin immunoblot confirms that equal amounts of protein were used in the analysis. D. Immunofluorescence of control and curcumin-treated DAOY cells (16 hours) for cleaved capase-3 (green) and F-actin (red). Bar, 20 μm.

To investigate the effect of curcumin on medulloblastoma, we treated the human medulloblastoma cell line DAOY with increasing concentrations of curcumin. After 16 hours, curcumin-treated DAOY cells underwent morphological changes, such as cell shrinking, rounding, and detachment (Figure 1A), suggesting that curcumin might induce cell death. Increasing concentrations of curcumin correlated with an increase in lactate dehydrogenase (LDH) release at 24 hours (Figure 1B). At higher concentrations of curcumin, LDH release was observed after as early as 8 hours of treatment, suggesting that curcumin induces cell death in a time- and concentration-dependent manner in these cells.

Curcumin-treated cells showed increased cleavage of caspase-3 (Figure 1C) and its downstream substrate poly(ADP-ribose)polymerase (PARP) (Figures. 1C, D). Both are hallmarks of dose- and time-dependent apoptotic cell death when compared with results for vehicle-treated cells. Furthermore, curcumin-induced apoptosis was blocked by z-VAD-FMK, a potent inhibitor of caspases (Additional file 1), suggesting that curcumin induces caspase-dependent apoptosis in DAOY cells. Increased PARP cleavage was also observed in two other medulloblastoma cell lines, D431 Med and D283 Med (Additional file 2), indicating that curcumin triggers apoptosis in medulloblastoma cells.

### Curcumin induces cell cycle arrest at G2/M phase

**Figure 2:**
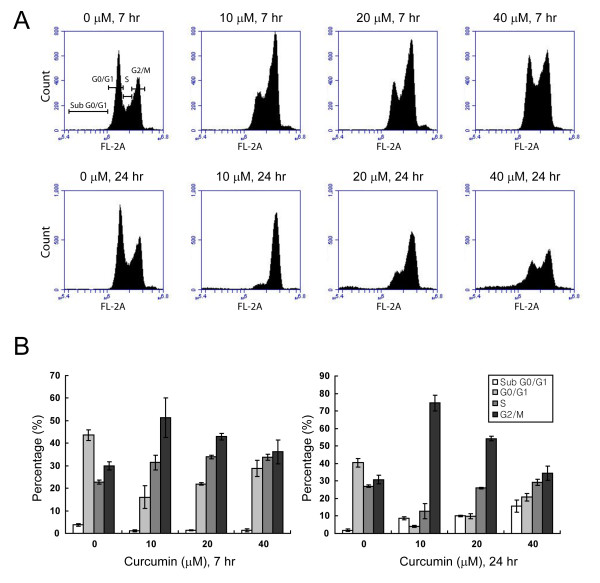
**Curcumin elicits G2/M phase arrest**. A. Cell cycle profiles of curcumin-incubated DAOY cells as analyzed by flow cytometry. Cells were exposed to curcumin at the indicated concentrations for 7 hours (top) and 24 hours (bottom). Note the increase in cells arrested in G2/M upon curcumin treatment. B. Quantitative analysis of cells in A obtained from three independent experiments. Averages of mean ± SD are shown.

Uncontrolled cell division can lead to programmed cell death. In carcinoma, it is well documented that curcumin can arrest cells either in the G1/S [35] or G2/M [36] stage of the cell cycle. We tested whether curcumin affects the cell cycle progression of DAOY cells using flow cytometry. DNA analysis of curcumin-treated cells revealed an increase of cells arrested in the G2/M phase as early as 7 hours after treatment (Figure 2A, B). Although in DMSO-treated control cells, only 29.9% of the cells were in G2/M phase, 51.4% and 42.9% of cells treated with 10 and 20 μM curcumin were found in G2/M, respectively. The effects of curcumin-induced cell cycle arrest were more pronounced after 24 hours of treatment, when 74.5% of curcumin-treated cells were in the G2/M phase compared with 30.8% of control cells. Thus, curcumin arrests DAOY cells at G2/M of the cell cycle. It is well accepted that a prolonged arrest in G2/M phase leads to apoptotic cell death [37,38]. Interestingly, with higher concentrations of curcumin, DAOY cells seemed to escape from cell cycle arrest, suggesting that high concentrations of curcumin could promote mitotic slippage and subsequent apoptosis.

### Curcumin induces acetylation of microtubules and microtubule-associated mitotic catastrophe

**Figure 3:**
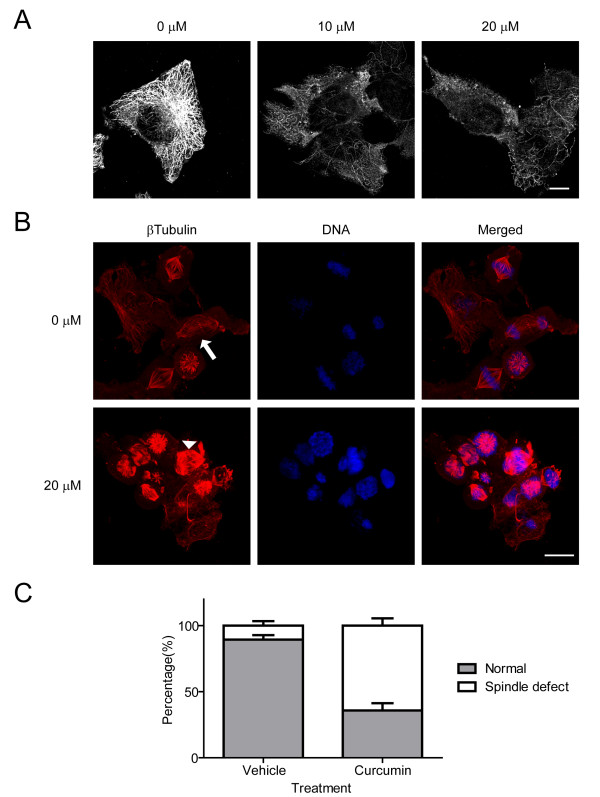
**Curcumin affects microtubule dynamics and mitotic spindle assembly**. A. Immunofluorescence for β-tubulin in DAOY cells treated with DMSO (0 μM) or curcumin (10, 20 μM) for 6 hours. Bar, 20 μm. B. Analysis of mitotic spindle microtubules in DAOY cells. Mitotic cells were released from the G2/M arrest in the presence or absence of curcumin. The cells were fixed after 60 min of release and stained for β-tubulin (red) and DNA (blue). The arrow indicates segregation of chromosomes along the mitotic spindle, while the arrowhead shows abnormal spindle formation and missegregation of chromosomes. Bar, 20 μm. C. Quantitative analysis of cells with abnormal mitotic spindles after 60 min of incubation with either vehicle or 20 μM curcumin. Error bars indicate standard deviations of three independent experiments.

It has been reported previously that curcumin inhibits microtubule assembly through binding with tubulin [39]. Thus, we hypothesized that curcumin-induced cell cycle arrest in G2/M might be due to its effects on microtubules and abnormal mitotic spindle formation. In interphase cells, we found a reduced microtubule density upon curcumin treatment (Figure 3A). However, the effect of curcumin on microtubules was much more pronounced in mitotic cells (Figure 3B). DAOY cells were arrested in prometaphase by a thymidine-nocodazole block and then released in the presence of curcumin or vehicle. Sixty minutes after release of the mitotic block, vehicle-treated cells clearly formed bipolar mitotic spindles and showed the alignment of compact chromosomes at the metaphase plate. Some cells showed segregation of chromosomes toward each pole (Figure 3B, arrow). Curcumin-treated mitotic cells exhibited a higher incidence of spindle abnormalities (64% ± 6%, compared with 11 ± 4% of vehicle control) and disorganized alignment of chromosomes (Figure 3B, arrowheads; and Figure 3C). These results suggest that curcumin preferentially affects the organization of spindle microtubules.

### Tubulin acetylation is increased in curcumin-treated medulloblastoma cells

**Figure 4:**
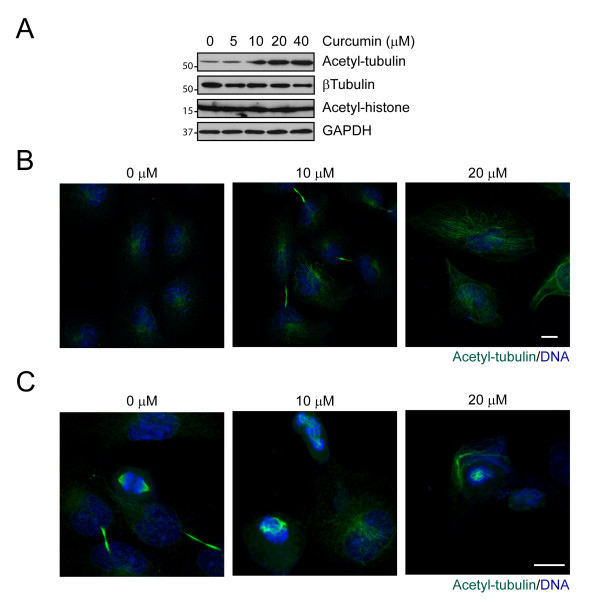
**Curcumin induces tubulin acetylation**. A. DAOY cells were exposed to increasing concentration of curcumin for 3 hours and then immunoblotted with antibodies indicated. Increased acetylation of tubulin was also observed in interphase (B) and mitotic (C) cells after 16 hours of curcumin treatment. Methanol-fixed cells were immunostained with antibody specific for acetylated tubulin (green). DNA stained blue. Bar, 20 μm.

Post-translational modifications of tubulin are crucial for regulating microtubule stability and function. Using modification-specific anti-tubulin antibodies, we found that in curcumin-treated DAOY cells, acetylated α-tubulin accumulated in a dose-dependent manner as early as 3 hours after treatment (Figure 4A). Similarly, curcumin increased α-tubulin acetylation in D431 Med and D283 Med cells (Additional file 2), while glutamylation and tyrosination were not affected in any of the medulloblastoma cell lines (data not shown). Interestingly, in interphase cells, acetylated α-tubulin was found predominantly in the perinuclear region of vehicle-treated cells, where the major population of stable microtubules resides (Figure 4B). In curcumin-treated DAOY cells, we found increased staining for acetylated α-tubulin throughout the cytoplasm. In addition, in mitotic DAOY cells, acetylated tubulin was found predominantly at the mitotic spindles and the intercellular bridge of cells undergoing cytokinesis (Figure 4C). In curcumin-treated cells, acetylated α-tubulin at the mitotic spindle pole was disorganized, suggesting that curcumin alters the acetylation pattern of microtubules and their organization at the spindle poles.

### Curcumin blocks HDAC activity

**Figure 5:**
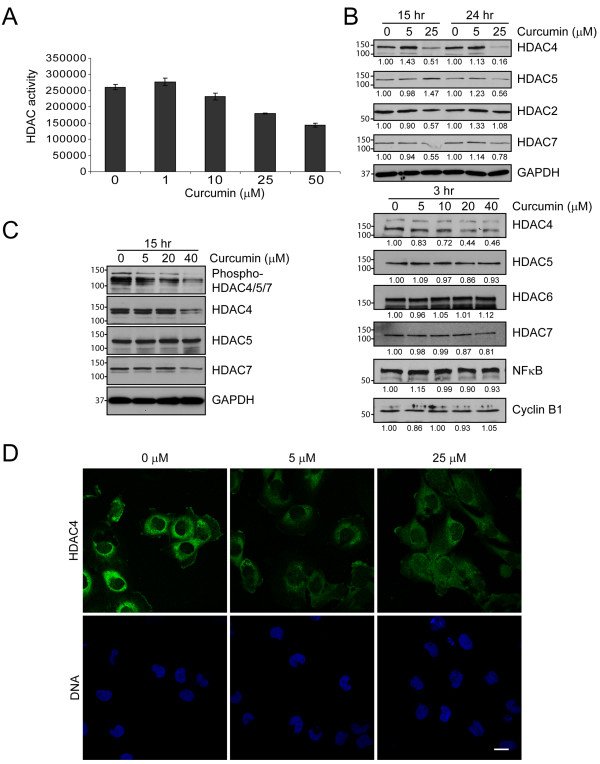
**Curcumin blocks HDAC activity**. A. Curcumin-treated DAOY cells (6 hours) were lysed and subjected to a fluoremetric HDAC activity assay as described in Materials and Methods. Data represent the average of the mean ± SEM of two independent experiments done in triplicates B. C. Expression levels of various HDAC family members (B) and phosphorylation level of HDAC4/5/7 (C) in DAOY cells treated with curcumin for 3, 15 and 24 hours. Representative immunoblots are shown. GAPDH immunoblots indicate loading of equal amounts of protein. Numbers below immunoblots in B are the mean of the intensity of three independent experiments, normalized to control cells at each time point and concentration. D. Subcellular localization of HDAC4 (green) in DAOY cells. Incubation with curcumin (6 hours) did not significantly affect the cellular distribution of HDAC4. Nuclei are indicated by DNA (blue). Bar, 30 μm.

The intricate balance between acetylation and deacetylation of proteins is regulated by the activities of HATs and HDACs. Using an *in vitro *activity assay, we found that increasing concentrations of curcumin blocked HDAC activity in DAOY cells (Figure 5A, 6 hours). To test whether curcumin affects a specific HDAC isoform, we screened the expression profiles of various HDAC family members upon curcumin treatment by immunoblotting. We detected several HDAC isoforms including HDAC2, 4, 5, and 7 in DAOY cells, but found only HDAC4 levels to be decreased upon curcumin treatment (Figure 5B), while other family members did not show any significant change. In addition, overall histone acetylation was not significantly altered in curcumin-treated cells (Figure 4A) suggesting that the observed reduction in HDAC activity might be due primarily to loss of HDAC4.

HDAC4 shuttles between the nucleus and cytoplasm, a process that is regulated by HDAC4 phosphorylation. While curcumin treatment dramatically reduced HDAC4 phosphorylation in all three medulloblastoma cell lines (Figure 5C, Additional file 3), the subcellular localization of HDAC4 did not change after six hours of curcumin treatment (Figure 5D, Additional file 4). Consistent with this notion, curcumin did not elicit changes in acetyl-histone levels in these cells (Figure 4A), suggesting that curcumin targets cytoplasmic HDAC4 and alters its function on cytoplasmic rather than nuclear substrates.

### Curcumin reduces medulloblastoma tumor growth *in vivo*

**Figure 6:**
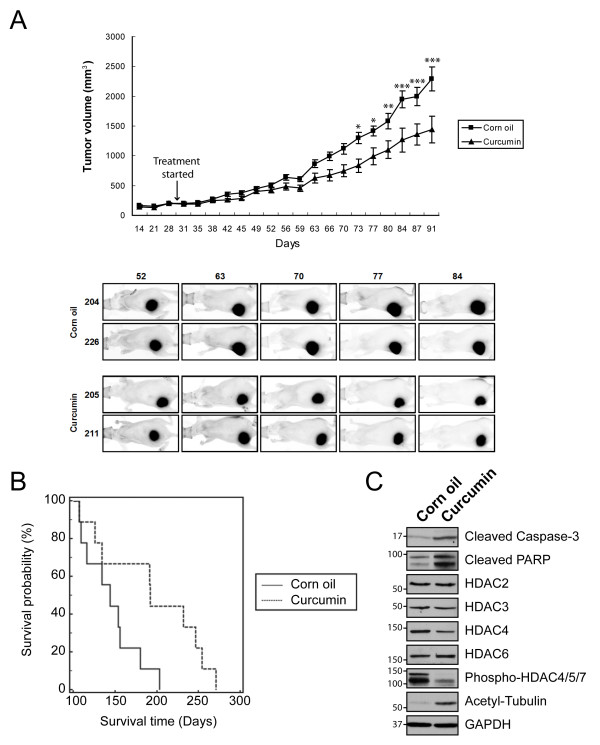
**Curcumin reduces tumor growth in DAOY *in vivo *tumor xenografts and Smo/Smo mice**. A. Effect of curcumin on tumor growth of subcutaneous xenograft tumors in nude mice. 30 days after cell injection (arrow), each group of mice (N = 12) received corn oil or curcumin (1 g/kg body weight) once daily and the tumor volume was measured by using a caliper. Data are expressed as mean ± SEM. *, P < 0.05; **, P < 0.01; ***, P < 0.001. Data are representative of three independent studies. Bottom panel, fluorescent ventral images of tumor-bearing mice. tdTomato-expressing tumors were imaged once a week and representative images are shown. B. Life span of control and curcumin-treated Smo/Smo (N = 9) plotted as Kaplan-Meier survival curve. The median survival time of curcumin-treated mice was 192 days vs 144 days of control mice (corn oil). P = 0.0308 (log rank analysis). C. Apoptotic markers, tubulin acetylation and HDAC expression and phosphorylation in tumor lysates obtained from each group of the Smo/Smo mice.

To evaluate the potency of curcumin to inhibit medulloblastoma growth *in vivo*, we used two independent mouse models: subcutaneous DAOY xenografts and the Smo/Smo transgenic medulloblastoma model. In Smo/Smo mice, a constitutively activated form of Smoothened (Smo) is expressed in CGNPs, resulting in a high tumor incidence with an early onset of medulloblastoma tumors [34]. DAOY cells stably expressing tdTomato were implanted subcutaneously, and curcumin was administered daily by oral gavage after tumors were established. As shown in Figure 6A and Additional file 5, curcumin suppressed the tumor growth significantly when compared with the control group (1442 ± 225 mm^3 ^in curcumin-treated group vs. 2294 ± 202 mm^3 ^in control group; P < 0.001). Fluorescence imaging of tumors established with tdTomato-DAOY cells confirmed the suppression of tumor growth by curcumin (Figure 6A, bottom panel).

One inherent problem of drug delivery for brain tumors is the BBB. Thus, we tested directly the efficacy of curcumin to inhibit tumor growth in brain tumors. Smo/Smo transgenic mice, a recently established medulloblastoma model, express the active mutant of Smo in CGNPs, and tumors form in more than 90% of mice within two months of age [34]. Curcumin was delivered orally once daily, and animals were monitored and sacrificed upon manifestation of clinical symptoms. As shown in Figure 6B, curcumin-treated mice had a significantly increased survival time when compared with corn oil-treated control mice, suggesting that curcumin can cross the BBB and exhibit therapeutic effects in the brain. Interestingly, the biochemical analysis of medulloblastoma tumors collected from each group showed an increase in apoptotic markers (cleaved caspase-3 and PARP), decrease in HDAC4 level and phosphorylation, and elevated acetylation of α-tubulin in curcumin-treated tumors when compared with control tumors (Figure 6C), mirroring the results obtained in cultured medulloblastoma cells.

## Discussion

In this study, we demonstrate that curcumin induces apoptosis in medulloblastoma cells and is accompanied by reduced HDAC4 expression, increased tubulin acetylation, and arrest at the G2/M phase of the cell cycle followed by mitotic catastrophe, and cell death. We also show anti-tumor effects of curcumin *in vivo *in tumor xenografts and a transgenic medulloblastoma tumor model. Thus, our *in vitro *and *in vivo *data suggest that curcumin has the potential to be developed as a therapeutic molecule for medulloblastoma.

Microtubules form the mitotic spindle during cell division. Because of the rapid assembly and disassembly of microtubules during the alignment and separation of chromosomes, spindle microtubules are in general more dynamic than interphase microtubules [40]. Compounds that inhibit these dynamics lead to cell cycle arrest in the G2/M phase, eventually resulting in cell death. Curcumin has been shown to bind to tubulin, to induce tubulin aggregation, and to depolymerize interphase and mitotic microtubules in HeLa and MCF-7 cells [39]. Consistent with these data, we observed reduced microtubule density in interphase medulloblastoma cells treated with curcumin. In mitotic cells, however, we found that while the mitotic spindle microtubules were disorganized, they displayed increased staining intensity, suggesting stabilization of microtubules. In addition, curcumin treatment of DAOY cells resulted in increased tubulin acetylation. Although the exact function of post-translational tubulin acetylation is not known, it is usually considered to be associated with increased microtubule stability [41]. Thus, it is possible that factors other than direct binding of curcumin to tubulin play a role in the altered organization of the mitotic spindle in curcumin-treated medulloblastoma cells.

We found that curcumin is a novel modulator of HDAC4. In curcumin-treated cells, HDAC activity was inhibited and HDAC4 expression was reduced, while the expression levels of other HDAC isoforms did not appear to be affected. At this point, we do not know how curcumin regulates HDAC4 expression and HDAC activity. Studies to determine the molecular mechanisms continue in our laboratory. Reduced HDAC activity and HDAC4 levels were observed as early as three hours upon curcumin-treatment, coinciding with increased α-tubulin acetylation. Mitotic spindles were altered as early as 30 min after treatment (data not shown) and very prominent after 60 min (Figure 3B), indicating a potential of curcumin as an anti-mitotic drug. At these early time points, we did not find any indication of curcumin-treated cells undergoing apoptosis, nor did we find substantial changes in some of the well-known signaling pathways affected by curcumin, such as NFκB (Figure 5B) or Akt (data not shown). Therefore, we suggest that HDAC4 inhibition in curcumin-treated cells might contribute to the induction of apoptosis rather than being a byproduct of apoptosis. This is further supported by our observation that inhibition of caspase-3 did not prevent reduced expression of HDAC4 upon curcumin treatment (Additional file 1). The effects of curcumin observed in cell lines were mirrored in *in vivo *models of medulloblastoma, namely DAOY xenografts and the Smo/Smo transgenic mice. In both medulloblastoma models, curcumin significantly reduced tumor growth and increased survival, respectively. Molecular analysis of curcumin-treated and control tumors revealed reduced HDAC4 expression and increased tubulin acetylation, suggesting that curcumin induces apoptosis by similar mechanisms in culture and *in vivo *medulloblastoma.

A disrupted equilibrium as a result of increased HDAC expression and activity has been associated with increased proliferation, migration, angiogenesis, differentiation, invasion, and metastasis and enables cancer cells to evade cell cycle arrest and apoptosis by suppressing the transcription of cell cycle inhibitors and pro-apoptotic factors [14,15,42]. Interestingly, a recent study found that forced expression of HDAC4 in cerebellar granule neurons protects these cells against apoptosis [43]. We show that curcumin targets HDAC4 in medulloblastoma cells and reduces HDAC activity. Thus, curcumin might target one of the critical pathways that allow cancer cells to evade apoptosis. Previous studies reported that curcumin represses p300/CBP HAT and inhibits acetylation of p53 [44,45]. However, we did not find changes in either p300 phosphorylation and histone H3 or p53 acetylation under our experimental conditions (data not shown), while HDAC4 expression was reduced in three medulloblastoma cell lines as well as *in vivo *(Figure 5B and Additional file 3). Similarly, studies in other experimental systems also found no effects of curcumin on p300 activity [44] suggesting that p300 inhibition by curcumin might be cell-type specific. Furthermore, we did not find significant changes in the levels of other HDAC isoforms, suggesting that in medulloblastoma cells HDAC4 is a specific target of curcumin.

In contrast to ubiquitous class I HDACs, HDAC4 as a class IIa family member is restricted to certain tissues, including the brain, and can shuttle between the cytoplasm and the nucleus. The translocation of HDAC4 from the cytoplasm to the nucleus is regulated by localization signals and interaction with 14-3-3 proteins through three conserved phosphorylation sites [46]. However, curcumin treatment did not alter the cytoplasmic localization of HDAC4 in DAOY cells, suggesting that curcumin's effect on HDAC4 might affect predominantly non-histone targets rather than chromatin structure and gene transcription. Interestingly, a recent study found that Shh signaling, a major signaling pathway affected in medulloblastoma, is regulated by Gli acetylation and HDAC1 [47]. Nevertheless, this study did not find any link between HDAC4 and Shh signaling in fibroblasts. However, given the cell-type specific expression pattern of HDAC4 we cannot exclude that such a link might exist in medulloblastoma cells. In addition, another study showed that curcumin inhibits the Shh pathway in medulloblastoma cells [32]. We found that curcumin was effective in the Smo/Smo medulloblastoma model, which increased survival, while HDAC4 expression was reduced at the same time. It remains to be determined whether HDAC inhibition is a missing link between curcumin and its effects on Shh signaling in medulloblastoma.

Although potential chemotherapeutics may show promise in medulloblastoma culture models, the BBB remains an obstacle for the development of drugs for brain tumors. Indeed, about 98% of all small molecule drugs and all large molecules such as therapeutic antibodies and peptides will be prohibited from crossing into the brain. We show that orally delivered curcumin increases survival in Smo/Smo mice and thus, exhibits chemotherapeutic effects in the brain. Our data are consistent with studies of curcumin in various central nervous system (CNS) disorders including Alzheimer's disease that showed a potent effect of orally delivered curcumin in the brain [9]. In addition, curcumin crossed the BBB and inhibited tumor growth in orthotopic glioblastoma models when administered through the tail vein [11] or injected i.p. [10]. Bioavailability of curcumin in the brain is further supported by multiphoton microscopic studies and radiolabel distribution studies in mice that showed that curcumin administered systemically can cross the BBB, can be absorbed in the brain, and exerts biological effects in the brain [7]. These studies are consistent with our observations that curcumin can cross the BBB, as manifested in increased survival in curcumin-treated Smo/Smo mice, and that curcumin is a valid anti-cancer agent for brain tumors.

Despite advances in treatment, a favorable outcome for patients with medulloblastoma lags behind many other pediatric cancers and is often associated with severe long-term side effects. For example, a small molecule inhibitor of Shh succeeded in eradicating spontaneous medulloblastoma in transgenic and transplantation mouse models [5]. However, while these agents might have no or limited side effects in adults, in juvenile mice even transient exposures to a Shh pathway inhibitor resulted in permanent defects in bone development [6]. In addition, while a first clinical trial was initially successful, the patient developed resistance within a short time [48,49] impeding its therapeutic potential against medulloblastoma. Thus, it remains a challenge to identify safer and effective drugs to treat pediatric brain tumors. Curcumin has been used as a spice for centuries in Asian cooking and has demonstrated its safety in phase I and II clinical trials in adults. No adverse reactions in clinical trials involving children have been reported so far [50,51]. Curcumin has potential anti-tumor effects in a variety of cancers including pediatric cancers such as osteosarcoma [52], neuroblastoma [53], and acute lymphoblastic leukemia [54]. Here, we report that curcumin induces apoptosis in medulloblastoma cells as well as *in vivo *models of medulloblastoma. While curcumin reduced tumor growth in tumor xenografts and increased survival in Smo/Smo mice, the tumors were not completely eradicated. A plethora of studies found that curcumin can potentiate the anti-tumor effects of other chemotherapeutics and irradiation. Thus, in combination with other modes of therapy, curcumin has the potential to develop into a therapeutic for medulloblastoma without the severe side effects found in current treatment regimens.

## Conclusions

Recently, curcumin has gained attention as a potent anti-cancer agent with no discernible side effects in several cancers. Our studies show that curcumin induces apoptosis in medulloblastoma cells, reduces tumor growth in medulloblastoma tumor xenografts and increases survival in Smo/Smo mice. Thus, curcumin has the potential to be developed as a therapeutic for medulloblastoma without the severe side effects found in current treatment regimens.

## Abbreviations

BBB: blood-brain barrier; CGNP: cerebellar granule neuron precursor; EGFR: epidermal growth factor receptor; HAT: histone acetyltransferase; HDAC: histone deacetylase; PARP: poly(ADP-ribose)polymerase; SHH: sonic hedgehog;

## Competing interests

The authors declare that they have no competing interests.

## Authors' contributions

SJL designed and performed the research, analyzed the data and drafted the manuscript; CK, VM, and PTF performed some of the research and analyzed the data; JMO provided the transgenic medulloblastoma mouse model and revised the manuscript critically for important intellectual content; SAR supervised the research, analyzed the data and drafted the manuscript. All the authors read and approved the final manuscript.

## Pre-publication history

The pre-publication history for this paper can be accessed here:

http://www.biomedcentral.com/1471-2407/11/144/prepub

## Supplementary Material

Additional file 1 **Curcumin-induced HDAC4 reduction and inhibiton of HDAC activity is not blocked by caspase inhibition**. A. DAOY cells were incubated for 2 hours with 20 μM z-VAD-FMK followed by curcumin treatment in the presence of inhibitor for an additional 8 hours. Lysates were prepared and immunoblotted for HDAC4, HDAC5, and cleaved caspase-3. GAPDH served as loading control. B. DAOY cells were treated with z-VAD-FMK and curcumin as described above and HDAC activity was measured by a fluoremetric HDAC activity assay. Data are representative of two independent experiments and the mean ± SD is shown. Click here for file

Additional file 2 **Curcumin induces apoptosis and tubulin acetylation in medulloblastoma cell lines**. D283 Med and D341 Med cells were incubated with different concentrations of curcumin for 24 hours, lysed and immunoblotted with cleaved PARP, acetyl tubulin and GAPDH antibodies, respectively. Click here for file

Additional file 3 **Curcumin reduces the expression and phosphorylation of HDAC4**. HDAC expression profiles in medulloblastoma cell lines. D283 Med (top panel) and D341 Med (bottom panel) cells were treated with increasing concentrations of curcumin for 24 hours and then subjected to immunoblotting with indicated HDAC antibodies. Click here for file

Additional file 4 **Curcumin does not affect subcellular localization of HDAC4**. DAOY cells were treated with curcumin or DMSO for 3 hours and then subjected to cytoplasm/nucleus fractionation. Equal amount of proteins from each fraction was subjected to immunoblotting for HDAC3, 4, 5 and phospho-HDAC4/5/7. Click here for file

Additional file 5 **Curcumin reduces tumor growth in DAOY *in vivo *tumor xenografts**. Effect of curcumin on tumor growth of subcutaneous xenograft tumors in nude mice. 30 days after subcutaneous injection of DAOY cells (arrow), corn oil or curcumin (1 g/kg body weight) were given to each group of mice (N = 8) once daily and the tumor volume was measured by using a caliper. Data are expressed as mean ± SEM. *, P = 0.0512; **, P = 0.0544; ***, P = 0.0694. Data are representative of two independent studies. Click here for file
