# Supplementary figures and images for: Curcumin-induced HDAC inhibition and attenuation of medulloblastoma growth in vitro and in vivo
Source: BMC Cancer. 2011 Apr 18;11:144. doi: 10.1186/1471-2407-11-144 (PMC3090367; doi:10.1186/1471-2407-11-144)

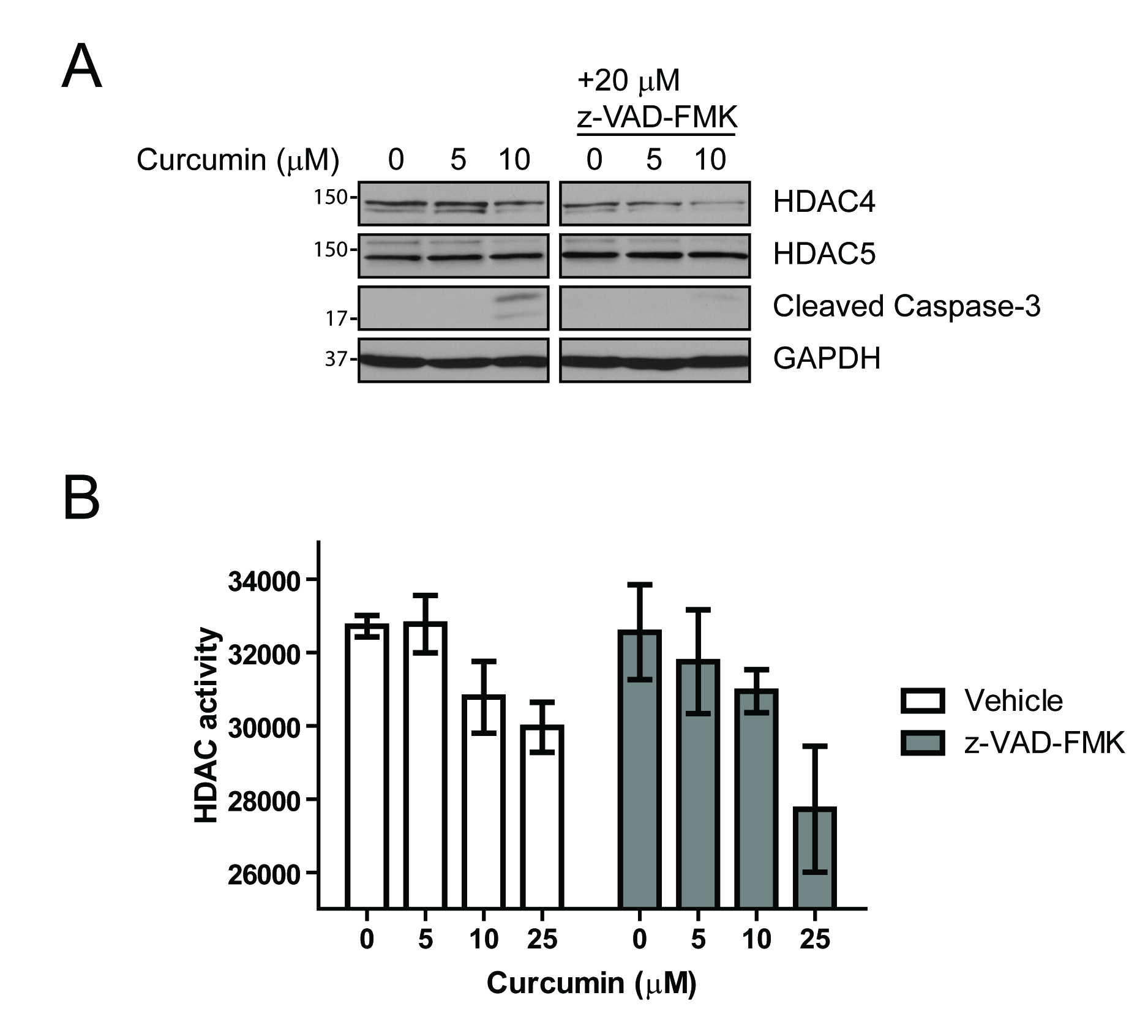

Supplement: Additional file 1 — Curcumin-induced HDAC4 reduction and inhibiton of HDAC activity is not blocked by caspase inhibition. A. DAOY cells were incubated for 2 hours with 20 μM z-VAD-FMK followed by curcumin treatment in the presence of inhibitor for an additional 8 hours. Lysates were prepared and immunoblotted for HDAC4, HDAC5, and cleaved caspase-3. GAPDH served as loading control. B. DAOY cells were treated with z-VAD-FMK and curcumin as described above and HDAC activity was measured by a fluoremetric HDAC activity assay. Data are representative of two independent experiments and the mean ± SD is shown. [file 1471-2407-11-144-S1.TIFF]

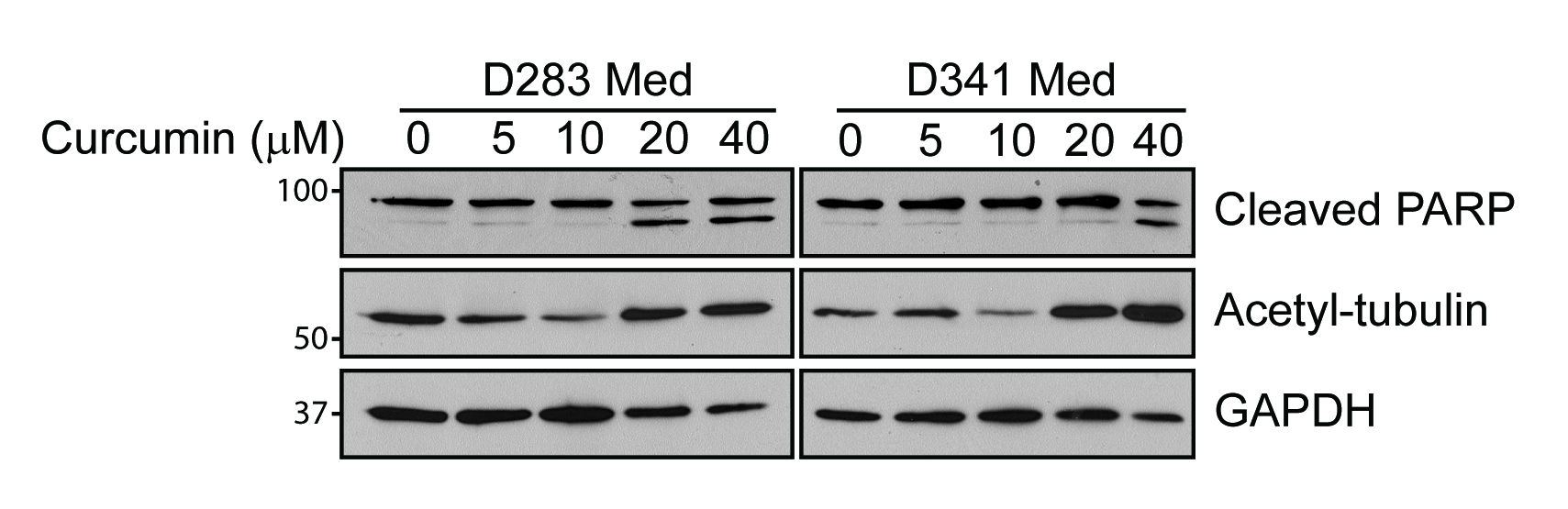

Supplement: Additional file 2 — Curcumin induces apoptosis and tubulin acetylation in medulloblastoma cell lines. D283 Med and D341 Med cells were incubated with different concentrations of curcumin for 24 hours, lysed and immunoblotted with cleaved PARP, acetyl tubulin and GAPDH antibodies, respectively. [file 1471-2407-11-144-S2.TIFF]

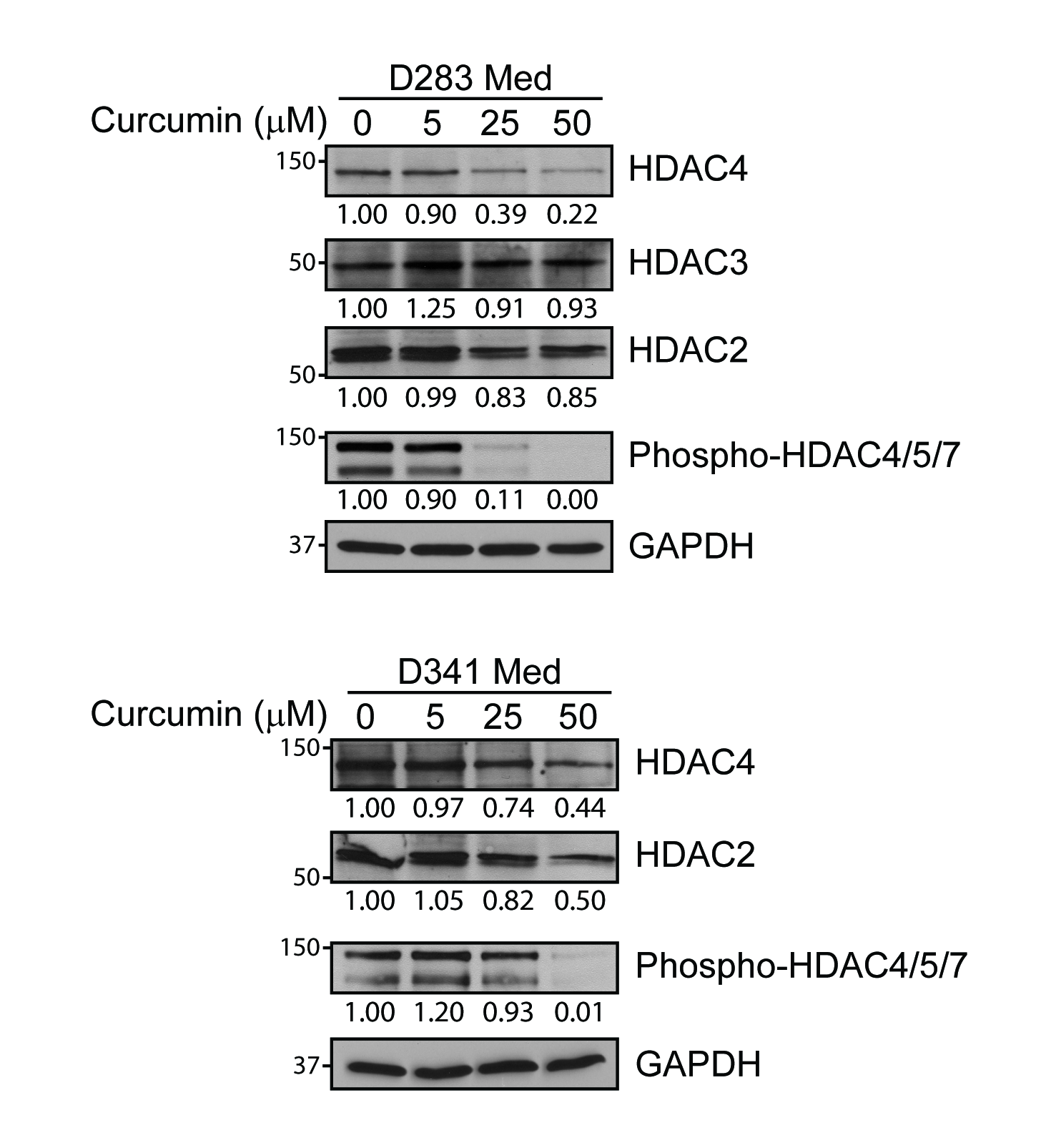

Supplement: Additional file 3 — Curcumin reduces the expression and phosphorylation of HDAC4. HDAC expression profiles in medulloblastoma cell lines. D283 Med (top panel) and D341 Med (bottom panel) cells were treated with increasing concentrations of curcumin for 24 hours and then subjected to immunoblotting with indicated HDAC antibodies. [file 1471-2407-11-144-S3.TIFF]

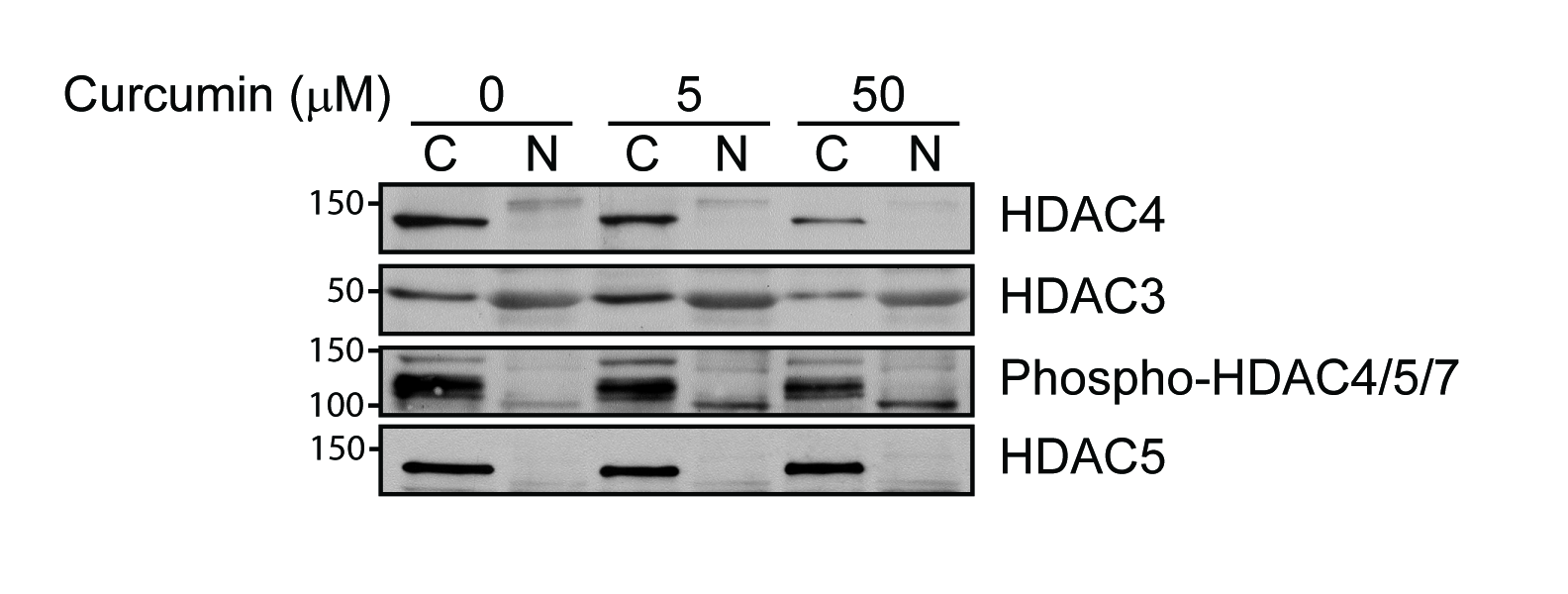

Supplement: Additional file 4 — Curcumin does not affect subcellular localization of HDAC4. DAOY cells were treated with curcumin or DMSO for 3 hours and then subjected to cytoplasm/nucleus fractionation. Equal amount of proteins from each fraction was subjected to immunoblotting for HDAC3, 4, 5 and phospho-HDAC4/5/7. [file 1471-2407-11-144-S4.TIFF]

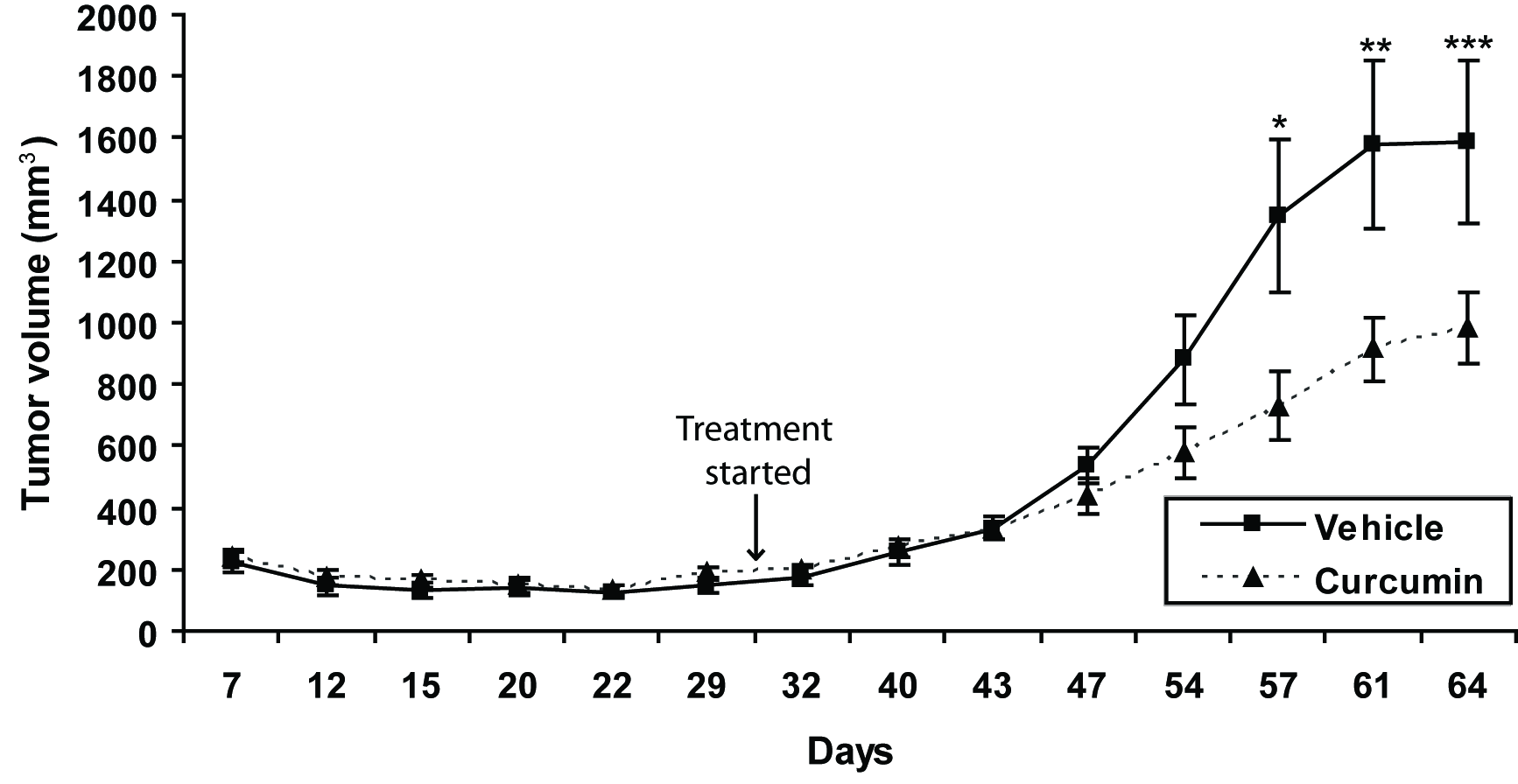

Supplement: Additional file 5 — Curcumin reduces tumor growth in DAOY in vivo tumor xenografts. Effect of curcumin on tumor growth of subcutaneous xenograft tumors in nude mice. 30 days after subcutaneous injection of DAOY cells (arrow), corn oil or curcumin (1 g/kg body weight) were given to each group of mice (N = 8) once daily and the tumor volume was measured by using a caliper. Data are expressed as mean ± SEM. *, P = 0.0512; **, P = 0.0544; ***, P = 0.0694. Data are representative of two independent studies. [file 1471-2407-11-144-S5.TIFF]
